# Advanced HyperSight™ imaging for patients with adaptive SBRT of prostate cancer: a longitudinal analysis of tissue demarcation

**DOI:** 10.1186/s13014-025-02730-8

**Published:** 2025-10-16

**Authors:** Ralf Schmidt, Thanh Nguyen, Alicia S. Bicu, Paula Cvachovec, Victor Siefert, Miriam Eckl, Marvin Willam, Matthias F. Froelich, Stefan O. Schoenberg, Michael Ehmann, Daniel Buergy, Sven Clausen, Jens Fleckenstein, Frank A. Giordano, Judit Boda-Heggemann, Constantin Dreher

**Affiliations:** 1 Department of Radiation Oncology, University Medical Center Mannheim, Medical Faculty Mannheim, University of Heidelberg, Theodor-Kutzer-Ufer 1-3, 68167 Mannheim, Germany; 2 DKFZ-Hector Cancer Institute, University Medical Center Mannheim, Mannheim, Germany; 3 Department of Radiology and Nuclear Medicine, University Medical Center Mannheim, Medical Faculty Mannheim, University of Heidelberg, Mannheim, Germany; 4 Mannheim Institute for Intelligent Systems in Medicine (MIiSM), Medical Faculty Mannheim, University of Heidelberg, Mannheim, Germany; 5 Junior Research Group “Intelligent Imaging for Adaptive Radiotherapy”, Mannheim Institute for Intelligent Systems in Medicine (MIiSM), Medical Faculty Mannheim, University of Heidelberg, Mannheim, Germany

**Keywords:** Adaptive radiotherapy, Stereotactic radiotherapy, HyperSight^TM^-CBCT, Prostate cancer, Ethos

## Abstract

**Background:**

Cone-beam computed tomography (CBCT)-based adaptive radiotherapy (ART) at the Ethos^®^ linear accelerator (eLinac) allows for daily anatomical and dosimetric adjustments and relies on robust image quality. This study evaluated the longitudinal image quality of the novel HyperSight^TM^-CBCT (hCBCT) compared to planning CT (pCT), using phantoms and data of prostate cancer patients undergoing adaptive stereotactic body radiotherapy (SBRT). Building on this, the longitudinal contour sharpness of the organs in fractional hCBCT and their usability for ART workflow across fractions was evaluated.

**Methods:**

Between December 2023 and May 2024, 26 prostate cancer patients receiving ART at the eLinac with hCBCT technology were enrolled. Phantom studies assessed pCT and hCBCT image quality. Patient based analyses of all 156 imaging scans (pCT and each of the fractional hCBCT) analyzed, longitudinally examined firstly image quality, secondly contour sharpness of prostate, seminal vesicles, and rectal wall, and thirdly confidence to delineate the structures for the ART workflow. Time required for the ART based parameters were recorded. Quantitative metrics included CT number changes in the fat adjacent to the prostate and seminal vesicles. Friedman’s test with Bonferroni correction, Spearman and Intraclass Correlation Coefficient (ICC) were used for statistics.

**Results:**

hCBCT scans showed robust image quality parameters in the phantom and patient based analysis across fractions. Inter-observer agreement was moderate, with lower rating score for resident compared to the experienced radiation oncologist (*p* < 0.001). Patient based analysis showed no significant differences of the contour sharpness of the prostate and seminal vesicles between pCT and initial hCBCT scan, but contour sharpness ratings declined across treatment fractions. Confidence for the delineation of prostate and seminal vesicles during ART was significantly decreased at later fractions (each p_adj_<0.05) and this correlated significantly with longer assessment times (p_adj_≤0.05). The CT attenuation of the fat tissue adjacent to the prostate and seminal vesicles significantly increased across the fractions (p_adj_<0.05).

**Conclusions:**

High-quality imaging for adaptive SBRT in prostate cancer is provided by hCBCT, which offers equivalent tissue visualization compared to pCT. Fraction-dependent decreases in contour sharpness can be detected using longitudinal hCBCT imaging. These decreases are likely related to treatment-induced tissue changes and may impact ART workflow. The rating of the observed effects may potentially be influenced by the observer’s experience.

## Introduction

The cornerstone of image-guided radiotherapy (IGRT) is three-dimensional (3D) imaging, such as cone-beam computed tomography (CBCT) systems integrated into linear accelerators. CBCT scans before irradiation ensure accurate patient and target positioning. However, compared to planning CT, CBCT image quality is often reduced due to hardware and software differences, including elevated noise levels and more artefacts [1, 2].

The frequent CBCT use in daily IGRT highlights the possibility to optimize high-precision radiotherapy by improving image quality. This may allow for monitoring treatment-related changes in tumoral and normal tissue, particularly in adaptive radiotherapy (ART). Online ART allows daily treatment plan adaptation based on anatomical changes and tumor responses observed in CBCT [3, 4]. This facilitates more precise radiation delivery to the target while sparing surrounding tissue [5, 6]. Consequently, ART holds a potential for advancing radiation oncology beyond IGRT, especially when paired with robustly high image quality to enable high-precision radiotherapy. Early clinical applications of ART have demonstrated improved target accuracy over IGRT [7]. By optimizing dose delivery to spare healthy tissue, ART shows great promise for high-precision radiotherapy, as exemplified in whole-neck irradiation for pharyngeal cancer [8].

The HyperSight™-CBCT (hCBCT) imaging system (Varian, Siemens Healthineers) was recently introduced on the ART-capable Ethos^®^ devices (eLinac). This advanced CBCT system features hardware and software improvements over conventional CBCT, including a larger detector and faster acquisition times, possibly enabling higher image quality with reduced noise and artefacts [9–12].

Given the critical role of imaging in ART, it is essential to extend pre-therapeutic technical evaluations and investigate the longitudinal performance and clinical application of hCBCT. The hCBCT based imaging has the potential to improve the detection of longitudinal anatomical tissue changes during the radiotherapy treatment course, and thus potentially serve as an image-guided monitoring approach if robust image quality is provided. Furthermore, the visualization of anatomical changes may have a direct impact on the ART workflow, in particular on the performance of adaptive target delineation.

Therefore, this observational study evaluated image quality and anatomic tissue changes in fractional hCBCT and pCT scans and the secondary impact on the ART workflow based on both pre-therapeutic phantom examinations as a standardized baseline and longitudinally in patients undergoing adaptive stereotactic body radiotherapy (SBRT) for prostate cancer, in order to assess the potential of hCBCT for this high precision radiotherapy approach.

## Patients and methods

### Study design and population

The study was conducted in two parts: Part A involved standardized phantom assessments of tissue visualization in CT imaging across two observers with varying levels of experience (one resident and one experienced radiation oncologist), forming the basis for Part B - a structured longitudinal evaluation of patient data for ART.

Part A: An inhomogeneous, intensity modulated radiotherapy (IMRT) 3D Pelvis Phantom (Sun Nuclear, Norderstedt; dimensions: 35.6 cm x 38.1 cm x 22.9 cm, Materials: Tissue and bone equivalent epoxy (Body and inserts)) and an Electron Density/Dual Energy Phantom (GAMMEX, Middleton) containing holes filled with inserts of various tissue and water equivalent materials were used for comparison of planning CT (pCT) scans (Brilliance BigBore, Philips) with and without metal artefact reduction (MAR) and hCBCT scans (pelvis protocol with iterative CBCT (iCBCT) and MAR reconstruction mode) at the eLinac (Table 1). All hCBCT scans were acquired using the Ethos^®^ platform on a Halcyon linear accelerator (Varian, Siemens Healthineers) with running software version 2.01.00. The “iCBCT Acuros reconstruction” was not used. Since the iCBCT algorithm was used with a hCBCT equipped device the CBCT scans investigated within this study were named generally as hCBCT.

Part B: Twenty-six patients with adaptive SBRT for primary prostate cancer were prospectively included in an IRB-approved observational registry study about ART (Ref:2023 − 557) at the Medical Faculty Mannheim, University of Heidelberg, from December 2023 to May 2024. Written informed consent was obtained.

The image quality of all 130 fractional hCBCT scan of ART at the eLinac was evaluated using patient-based measurements, compared to pCT scans of treatment planning. The hCBCT scans were acquired using the pelvis preset (with iCBCT reconstruction mode) with an acquisition time of 6 s. The MAR reconstruction mode was performed in three patients with femoral/hip implants.

The patients had a mean age of 75.8 ± 15.6 years at pCT.

The decision to use adaptive SBRT for prostate cancer was based on physician’s judgment. Institutional radio-oncologic criteria and planning parameters were based on the PACE trial [13]. For patient characteristics, target delineation and dose prescription see Table 2. Margin variations were allowed based on physician’s discretion. Additionally, androgen deprivation therapy (ADT) was administered based on the physician’s judgment, depending on individual risk factors. One patient initiated new ADT during treatment.

Initial treatment planning based on pCT was carried out within the Ethos^®^ Appliance System (Varian, Siemens Healthineers). Fractional adaptive treatment planning was performed using hCBCT for contouring, followed by a dose calculation of an adapted treatment plan for each fraction on a deformed synthetic CT scan out of hCBCT and pCT.

**Table 1:** Details of imaging protocols used for clinical application on pCT (planning computed tomography (CT) scanner: Brilliance BigBore, Philips) with and without metal artefact reduction (MAR), HyperSight™ CBCT imaging (pelvis protocol) with iCBCT and MAR reconstruction mode at the Ethos linear accelerator (Varian, Siemens Healthineers). Displayed are parameters for peak kilovoltage (kVp), Milliampere-seconds (mAs), slice thickness, pixel size and CT dose index-volume (CTDIvol). For mAs and CTDIvol the mean value ± standard deviation (SD) is displayed, in case of pCT with variable parameters as compared to CBCT with rigid parameters

Details of imaging protocols					
Imaging Mode	kVp	mAs (mean ± SD)	Slice thickness (mm)	Pixel size (mm)	CTDIvol (mGy) (mean ± SD)
pCT	120	175.2 ± 49.8	2	1.0507 × 1.0507	13.6 ± 15.5
pCT MAR	120	182.0 ± 56.6	2	1.0507 × 1.0507	10.8 ± 3.7
HyperSight™ iCBCT	125	468	2	1.0507 × 1.0507	8.9
HyperSight™ MAR	125	468	2	1.0507 × 1.0507	8.9

**Table 2:** Patient characteristics and general prescription details for adaptive radiotherapy (ART). The ART treatment concept was determined based on the assigned risk group with dose prescriptions to the clinical target volume (CTV), planning target volume (PTV), and in case of unfavourable intermediate/high risk to a second PTV. Margin variations were allowed based on physician’s discretion

Risk group according to D’amico [14]	Number	ART Dose prescription for 5 fractions
low	4	*Low/favourable intermediate risk*:
Intermediate (favourable and unfavourable)	20	36.25 Gy to the PTV = (prostate + proximal 1 cm of seminal vesicles) + 4–6 mm 40 Gy to a simultaneous boost region defined as the CTV = (prostate + proximal 1 cm of seminal vesicles) + 2 mm
		*Additional for Unfavourable intermediate/high risk*: 30 Gy to a 2nd PTV = (prostate + proximal 2 cm of seminal vesicles) + 4–8 mm
high	2	

### A: Phantom imaging assessment

**Fig. 1:**
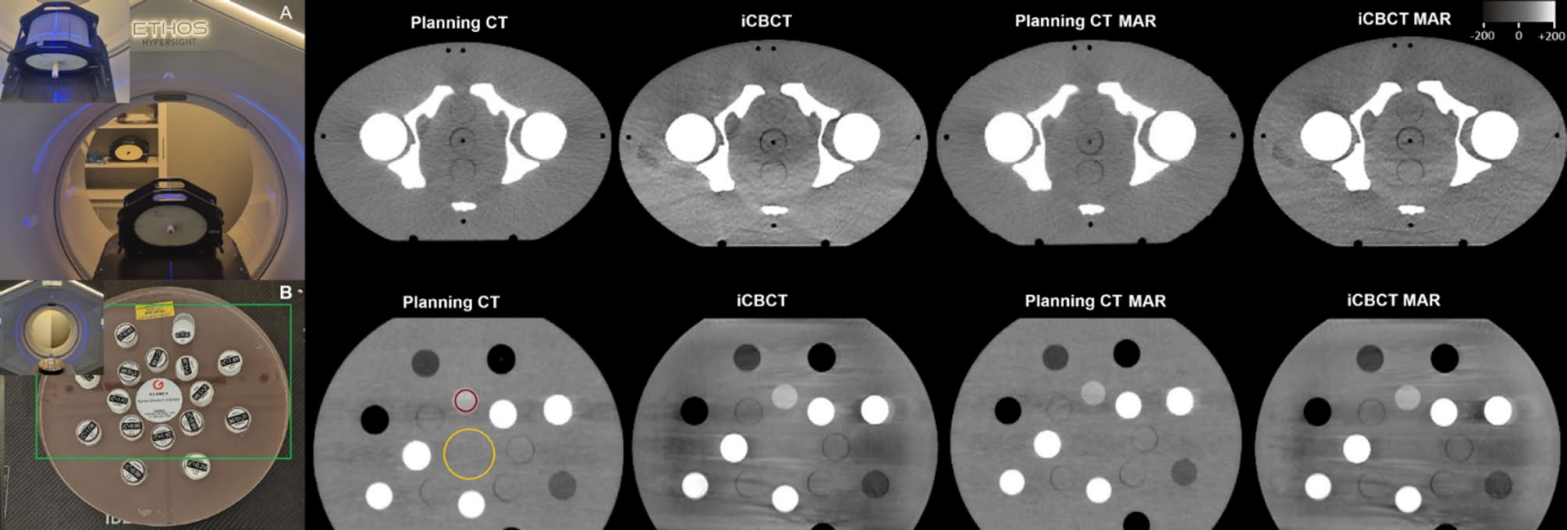
HyperSight™ phantom imaging. The imaging protocol used two test objects: (**A**) an inhomogeneous IMRT 3D Pelvis Phantom (Sun Nuclear) and (**B**) an Electron Density/Dual Energy Phantom (GAMMEX). All scans were acquired using both conventional planning computed tomography (CT) (Brilliance BigBore, Philips) with and without metal artefact reduction (MAR) and the investigational HyperSight™ conebeam CT (CBCT) system on an Ethos^®^ linear accelerator (Varian, Siemens Healthineers), with pelvis protocol and iCBCT, and MAR reconstruction. Quantitative image analysis focused on specific regions of interest (ROIs) within the electron density phantom: ROI 1 (yellow) assessed general tissue parameters for contrast and contrast-to-noise ratio calculations, while ROI 2 (red) evaluated liver-equivalent tissue characteristics including Signal-to-Noise-Ratio (SNR) and coefficient of variation. Enlarged views of key regions are highlighted in the green boxed area

The phantom scan setup is displayed in Fig. 1.

Overall image quality was assessed in the 3D pelvic phantom by two radiation oncologists (observers R1 = CD and R2 = RS, with 8 and 4 years of abdominopelvic CT imaging experience, respectively) with windowing set to width: 400, level: 0 Hounsfield Unit (HU). Both observers, unblinded to the three image setups, independently assigned ratings for the entire phantom for three horizontal slice heights (top, middle, low) based on a 5-point Likert scale and calculated average scores [15].

- Overall Image quality (rating of streak, beam hardening, windmill, metal artefacts): 1 = Major artefacts. 2 = Marked artefacts. 3 = Moderate artefacts. 4 = Minor artefacts. 5 = No artefacts.
- Edge Sharpness of tissue and bones: 1 = No clear edges. 2 = Poor edge definition. 3 = Moderate edge sharpness. 4 = Clear edges. 5 = Well defined edges.

Quantitative image quality parameters were determined for the Electron Density Phantom using contoured Regions of Interest (ROI) (in consensus for R1 and R2) (for ROI placement see Fig. 1). A liver-equivalent inlay with a Phantom Weighted Error (P_e_^w^) of 1.06 was chosen as the source for tissue signal. To calculate contrast-values, the central region of the phantom without inlays was chosen. For each of the three image setups three slices were evaluated (top, middle, low). On these slices a circular ROI within the inner border of the inlay of approximately 2.5 cm diameter was placed and average mean values and standard deviations (SD) were calculated. Analysis included calculating the Signal-to-Noise-Ratio (SNR), Contrast (C), Contrast-to-Noise-Ratio (CNR), and Coefficient of Variation (CV) with following formulas.

$$\rm{SNR\,=\,\frac{\text{M}ean\_\text{L + 1000}}{\text{SD}\_\text{L}},\quad C\,=\,\text{Mean}\_\text{L}-\text{Mean}\_\text{C},}$$

$$\rm{CNR\,=\,\frac{2\times{\left(\text{M}\text{e}\text{a}\text{n}\_\text{L}-\text{M}\text{e}\text{a}\text{n}\_\text{C}\right)}^{2}}{{\text{SD}\_\text{L}}^{2}+{\text{SD}\_\text{C}}^{2}}\, and}$$

$$\rm{CV\,=\,\frac{\text{SD}\_{L}}{\text{M}\text{e}\text{a}\text{n}\_\text{L}}\text{*}100\ \%}$$

where Mean_L/SD_L and Mean_C/SD_C are the means/SD of the liver-equivalent inlay (_L) and the central region of the phantom (_C). To align HU with tissue attenuation properties for different scanners, + 1000 HU was added to Mean_L within the SNR formula. A non-linear CNR calculation was used to enhance sensitivity to detectability differences in low-contrast tissues [16].

To test longitudinal stability, the phantom was scanned a second time at a different time point. The measurements and assessments were then repeated and compared.

### B: Qualitative patient based imaging assessment

Both observers conducted independent qualitative imaging analyses of pCT and fractional hCBCT scans from fractions 1–5 (fx1-5) in chronological order and in separate, limited sessions. The scans were compared side-by-side based on a 5-point Likert scale after blanking out of the delineated contours of the applied ART [15]:

First, both observers assessed overall image quality, and then subjective imaging contrasts for tissue differentiation/contour sharpness of different structures:

- Overall Image quality (rating of streak, beam hardening, windmill, metal artefacts): 1 = Major artefacts. 2 = Marked artefacts. 3 = Moderate artefacts. 4 = Minor artefacts. 5 = No artefacts.
- Tissue differentiation of pelvic organs and structures: 1 = No structure identifiable. 2 = impaired differentiation. 3 = moderate differentiation. 4 = Good differentiation. 5 = excellent differentiation.
- Contour sharpness of the prostate, seminal vesicles, and rectoprostatic border/rectal wall: 1 = no contour differentiation. 2 = impaired contour sharpness. 3 = moderate contour sharpness. 4 = good contour sharpness. 5 = excellent contour sharpness.

Second, the two observers evaluated the usability of the imaging scan for adaptive treatment planning and the confidence of structure delineation as in a typical ART workflow:

- Usability of the imaging for ART: 1 = not usable. 2 = impaired usability. 3 = moderate usability. 4 = good usability. 5 = excellent usability.
- Confidence to delineate the target structures prostate/seminal vesicles, and organs at risk for ART: 1 = No confidence. 2 = Considerably impaired confidence. 3 = Low confidence. 4 = Good confidence. 5 = High confidence.

The time required for rating the ART related assessments per scan was recorded for both observers.

### B: Quantitative patient based imaging assessment

The quantitative parameters were determined in chronological order, in limited sessions, and by consensus for R1 and R2.

**Fig. 2:**
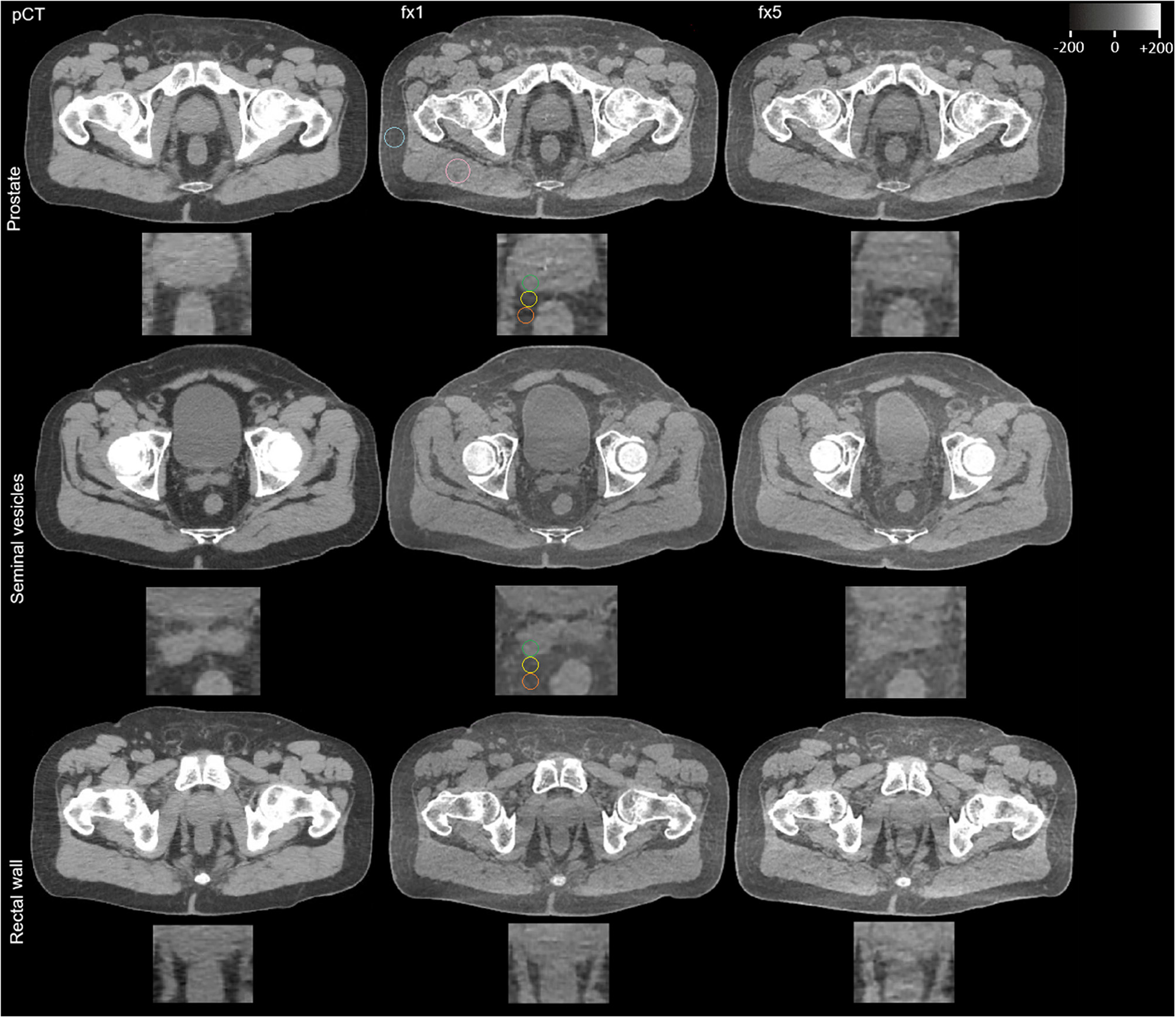
Comparison of Pelvic Imaging. Shown is the comparative analysis of pelvic imaging quality across treatment fractions. The planning computed tomography (pCT) scan (Brilliance BigBore, Philips) serves as the baseline for evaluating HyperSight™ CBCT scans on an Ethos^®^ linear accelerator (Varian, Siemens Healthineers) from the first (fx1) and fifth (fx5) fractions, with particular focus on the prostate-seminal vesicle complex and anterior rectal wall. Following key findings emerge: First, while pCT and fx1 CBCT show comparable contour sharpness, there is a noticeable decline in contour sharpness with fx5, particularly at the prostate-rectal interface. This trend is quantified by strategically placed regions of interest (ROIs) at tissue interfaces at the height of both target regions prostate and seminal vesicles (ROI1 = green, outer organ tissue/ROI2 = yellow, fat at the organ border/ROI3 = orange, peripheral fat) (see Results Sect. “Quantitative patient based analysis: longitudinal change of CT Attenuation in fractional hCBCT at the target interface”). Structural reference ROIs (turquoise/pink) in pelvic fat and gluteus maximus muscle are used to calculate the quantitative parameters of image quality (see Results Sect. “Quantitative patient based analysis: image quality parameters”)

First, quantitative image quality parameters were assessed of pCT and fractional hCBCT including the SNR, C, CNR, and CV with following formulas.

$$\rm{SNR\,=\,\frac{\text{M}ean\_\text{M + 1000}}{\text{SD}\_{M}},\quad C\,=\,\text{Mean}\_{M}-\text{Mean}\_{F},}$$

$$\rm{CNR\,=\,\frac{2\times{\left(\text{M}\text{e}\text{a}\text{n}\_\text{M}-\text{M}\text{e}\text{a}\text{n}\_\text{F}\right)}^{2}}{{SD\_M}^{2}+{SD\_F}^{2}}\:and}$$

$$\rm{CV\,=\,\frac{\text{SD}\_{M}}{\text{M}\text{e}\text{a}\text{n}\_\text{M}}\text{*}100\ \%}$$

where Mean_M/SD_M and Mean_F/SD_F are the means/SD of the gluteal muscle (_M) and pelvic fat (_F) (for ROI placement see Fig. 2). The tissues were chosen due to low dose exposure and the possibility of robust ROI placements. To align HU with tissue attenuation properties for different scanners, + 1000 HU was added to the mean signal within the SNR formula.

Second, quantitative assessment of longitudinal changes in CT attenuation at the target interface (at the level of both regions prostate and of the seminal vesicles) included the analysis of the mean CT numbers in three different ROIs for both regions on fractional hCBCT (for ROI placement see Fig. 2): within the target structure (peripheral part of the prostate or seminal vesicle; ROI1), directly adjacent to ROI1 (within the proximal 1 cm of surrounding fat; ROI2) and in the fat tissue directly adjoining ROI2, located 1–2 cm from the target (ROI3). Delta values of mean CT numbers ROI1-ROI2 and ROI2-ROI3 were calculated.

### Statistical analysis

Calculation of quantitative imaging parameters was based on MATLAB R2024b (The MathWorks; Natick, MA, USA), statistical analysis was performed with R (version 4.4.1, R Foundation for Statistical Computing, Vienna, Austria). Inter-observer agreement was evaluated using the Intraclass Correlation Coefficient (ICC) with an average measures fixed-raters model. ICC thresholds were: <0.50 (poor), 0.50–0.75 (moderate), 0.75–0.90 (good), and ≥ 0.90 (excellent). Significant differences between observers were tested by Wilcoxon-signed rank test. Qualitative and quantitative imaging parameters of pCT and fractional hCBCT scans were compared using the Friedman’s test. Correlations were tested by Spearman Correlation Coefficient (CC). Significance was set at *p* < 0.05. Post-hoc analysis with Bonferroni correction reported adjusted p-values (p_adj_).

## Results

### Phantom based analysis

Both observers rated MAR and iCBCT hCBCT scans consistently with a mean score of 4 for all parameters. For pCT images (both with and without MAR), mean scores for overall image quality and tissue differentiation were 4.3/5 (R1) and 4.3/4.7 (R2). The ratings of the two scans taken at different time points were not significantly different (each p_adj_>0.05). Quantitative parameters are detailed in Table 3. The quantitative parameters of the two scans taken at different time points were not significantly different (each p_adj_>0.05).

**Table 3:** Quantitative parameters of the electron Density/Dual energy Phantom (GAMMEX, Middleton) assessment for two different time points (t1, t2): shown are values of mean ± standard deviations from two independent Phantom scans, each for planning computed tomography (pCT, at brilliance BigBore, Philips) with and without metal artefact reduction (MAR), HyperSight^TM^–conebeam computed tomography (CBCT) pelvis protocol with iCBCT and MAR reconstruction mode on an Ethos^®^ linear accelerator (Varian, Siemens Healthineers). Quantitative parameters are derived from measurements of two different regions of interest with different CT Attenuation characteristics: Signal-to-Noise-Ratio (SNR), contrast (C), contrast-to-noise-ratio (CNR) and coefficient of variation (CV). The values for C are given in HU, for CV in %, while the remaining parameters have no unit

Quantitative phantom assessment								
	pCT		pCT MAR		HyperSight™ iCBCT		HyperSight™ MAR	
	t1	t2	t1	t2	t1	t2	t1	t2
SNR	108.4 ± 10.1	120.9 ± 7.4	155.5 ± 25.2	156.2 ± 8.7	165.5 ± 62.5	160.4 ± 15.0	127.3 ± 29.1	123.6 ± 39.2
C	78.2 ± 16.0	82.6 ± 3.8	66.9 ± 3.6	66.3 ± 2.9	80.1 ± 23.2	91.1 ± 3.1	82.4 ± 14.2	78.7 ± 1.8
CNR	77.4 ± 42.6	101.5 ± 13.6	97.9 ± 34.2	124.0 ± 22.1	60.1 ± 25.7	75.3 ± 14.8	69.9 ± 38.8	65.4 ± 28.5
CV	13.7 ± 2.9	12.0 ± 0.9	11.05 ± 1.8	11.39 ± 0.9	10.6 ± 5.8	8.4 ± 1.0	12.0 ± 3.3	12.5 ± 4.0

### Patient characteristics

Twenty-six prostate cancer patients with 156 imaging scans were included for analysis. Figure 2 illustrates a longitudinal course of pCT and hCBCT scans during ART for SBRT.

### Qualitative patient based analysis: image quality and tissue differentiation

Analysis of overall image quality showed robustly high values with no significant differences between fx1-5. R1 rated pCT significantly higher than fx1-5 (each p_adj_≤0.05) while the ratings of R2 showed no significant differences between pCT and fx1-5.

**Fig. 3:**
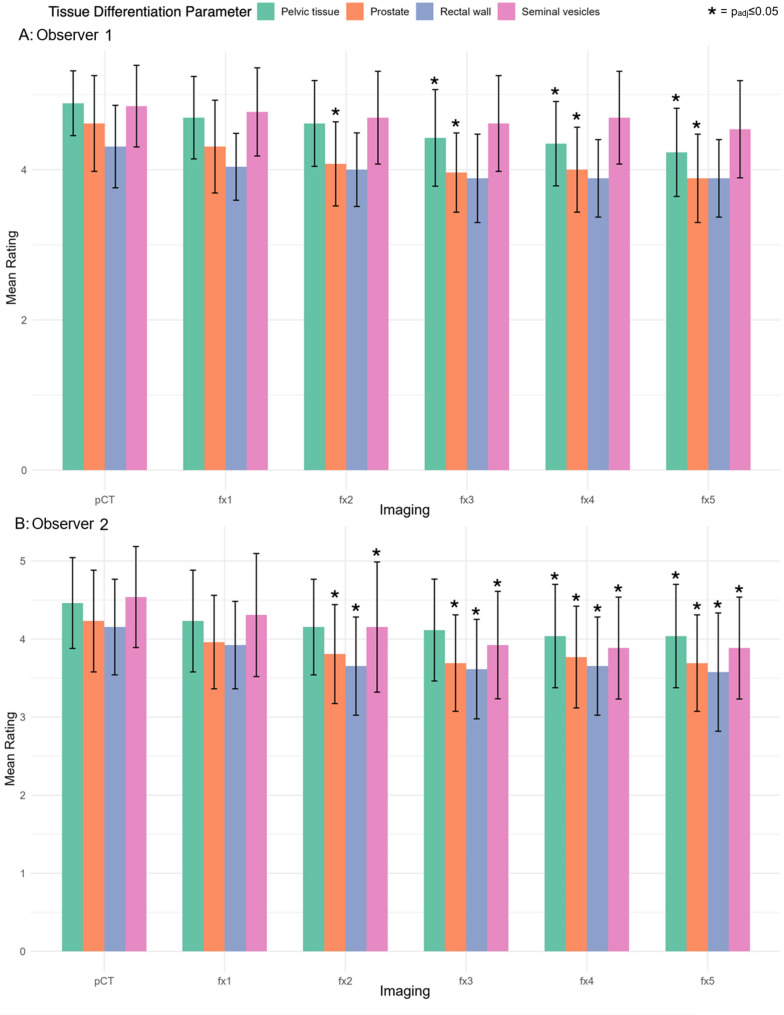
Mean Ratings of qualitative tissue differentiation parameters. Shown are Likert-scale ratings of computed tomography (pCT) scans (Brilliance BigBore, Philips) and fractional HyperSight™ CBCT scans on an Ethos^®^ linear accelerator (Varian, Siemens Healthineers) for adaptive SBRT for prostate cancer with different colours (parameters: pelvic tissue differentiation, contour sharpness of the prostate, rectal wall and seminal vesicles) for Observer R1 (**A**) and Observer R2 (**B**) with error bars representing standard deviations. Asterisks indicate statistical significance in the post-hoc comparison of qualitative imaging parameters (p_adj_<0.05) between pCT and one of the five fractional HyperSight^TM^-CBCT scans (fx1-5) during adaptive SBRT for prostate cancer

Regarding the rating of tissue differentiation and contour sharpness, Fig. 3 presents the longitudinal decrease of the ratings from pCT to fx5 for both observers. The inter-observer agreement showed moderate agreement (ICC = 0.73) with the scores of the two observers being significantly different (mean ± SD: 4.3 ± 0.7 for R1 vs. 4.0 ± 0.7 for R2, *p* < 0.001).

For the parameter tissue differentiation, R1 rated pCT significantly higher compared to fx3-5 (each p_adj_≤0.05). R2 rated pCT significantly higher compared to fx4-5 (each p_adj_≤0.05). For contour sharpness of the prostate, significant differences were observed for R1 and R2 with a higher pCT score compared to fx2-5 (each p_adj_≤0.05). For contour sharpness of the seminal vesicles, no significant differences were observed in post-hoc comparisons for R1. For R2 pCT was rated significantly higher compared to fx2-5 (each p_adj_≤0.05). For contour sharpness of the rectal wall, no significant differences were found for R1. R2 rated pCT significantly higher compared to fx2-5 (each p_adj_≤0.05).

Post-hoc comparisons between pCT and fx1 and other comparisons were not significantly different for both observers.

### Qualitative patient based analysis: usability and confidence of delineation for ART

**Fig. 4:**
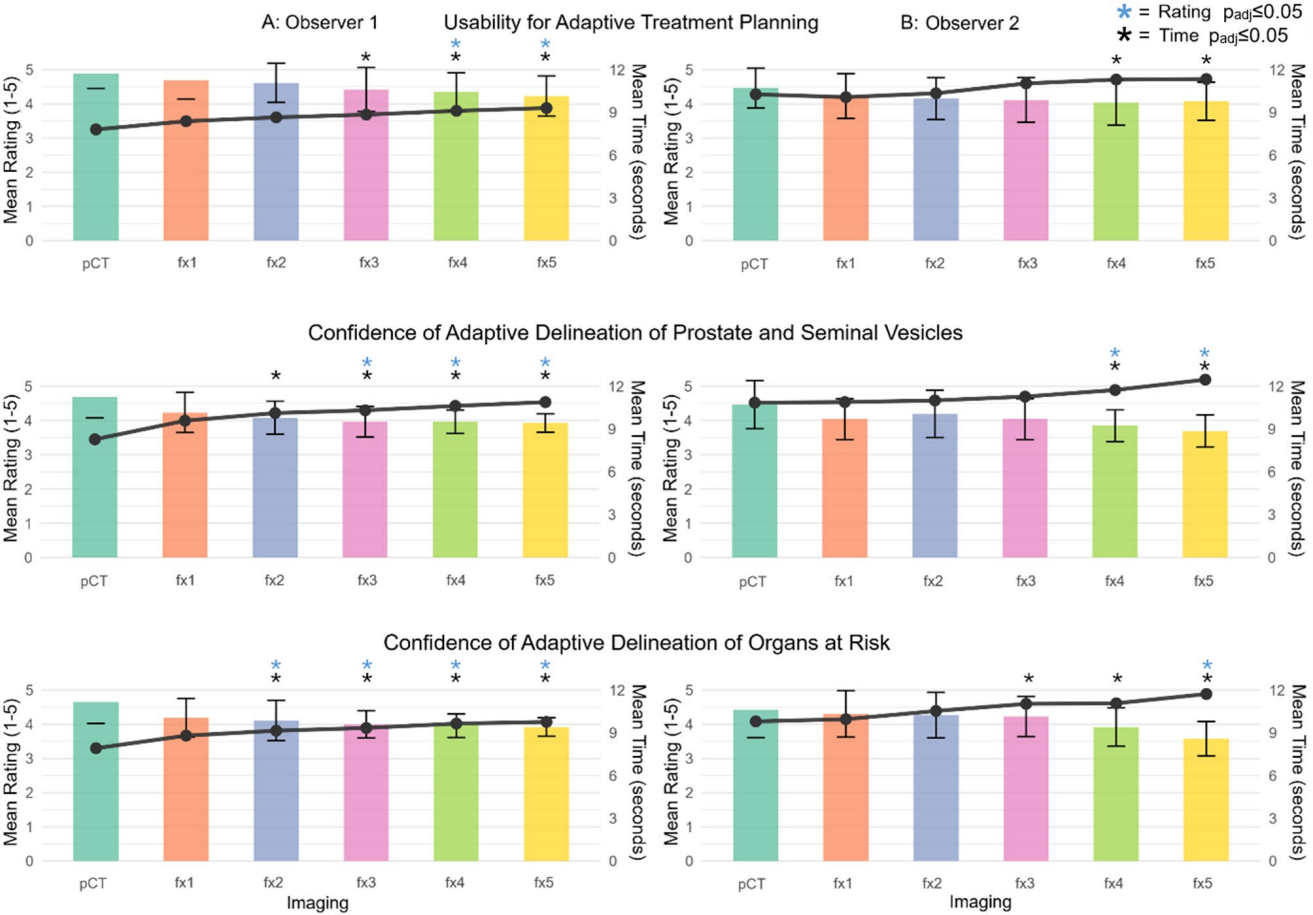
Mean Ratings of qualitative imaging parameters for usability and confidence of delineation for adaptive treatment planning. Shown are Likert-scale ratings of computed tomography (pCT) scans (Brilliance BigBore, Philips) and fractional HyperSight™ CBCT scans on an Ethos^®^ linear accelerator (Varian, Siemens Healthineers) for adaptive SBRT for prostate cancer (parameters: usability for adaptive treatment planning, confidence of adaptive delineation of prostate/seminal vesicle target structures and the organs at risk) for Observer R1 (**A**) and Observer R2 (**B**) with error bars representing standard deviations. A black time curve is overlaid on each of the plots to indicate the mean duration of each of the rating assessments. Blue (rating) and black (time) asterisks indicate statistical significance in the post-hoc comparison of the rated parameters and the time consumption (p_adj_<0.05), respectively, between pCT and one of the five fractional HyperSight^TM^-CBCT scans (fx1-5) during adaptive SBRT for prostate cancer

The parameters usability for ART and confidence of delineation for adaptive treatment planning longitudinally decreased for both observers from pCT to fx5 (both *p* < 0.001), while the time required for assessment increased longitudinally (Fig. 4). The inter-observer agreement showed moderate agreement (ICC = 0.67) with the scores of the two observers being significantly different (mean ± SD: 4.3 ± 0.6 for R1 vs. 4.1 ± 0.7 for R2, *p* < 0.001).

The confidence for delineation of seminal vesicles and prostate demonstrated significantly decreased ratings with fx3-5 for R1, and fx4-5 for R2 compared to pCT (each p_adj_≤0.05). The confidence for organs at risk delineation ratings were significantly decreased with fx2-5 for R1 and fx5 for R2 compared to pCT (each p_adj_≤0.05).

Assessment times of the ART related parameters were longitudinally significantly different for both observers (each *p* < 0.001). Assessment time was significantly shorter with pCT compared to fx3-5 and fx2-5 for R1 (each p_adj_≤0.05) across the parameters of overall usability and confidence of delineation. R2 demonstrated significantly shorter assessment times with pCT compared to fx4-5 for overall usability, prostate/seminal vesicle delineation, and compared to fx3-5 for organs at risk delineation (each p_adj_≤0.05).

Post-hoc comparisons between pCT and fx1 and the other comparisons were not significantly different for both observers.

The time required to assess the three parameters usability for ART delineation confidence of the prostate and seminal vesicles and of the organs at risk for complete image scan correlated significantly with the fractional numbers from fx1-5 for all ART related parameters (R1: Spearman’s CC = 0.89/0.97/0.86; *p* = 0.02/0.002/0.029, R2: Spearman’s CC = 0.95/0.95/0.79; *p* = 0.056/0.007/0.007).

### Quantitative patient based analysis: image quality parameters

**Fig. 5:**
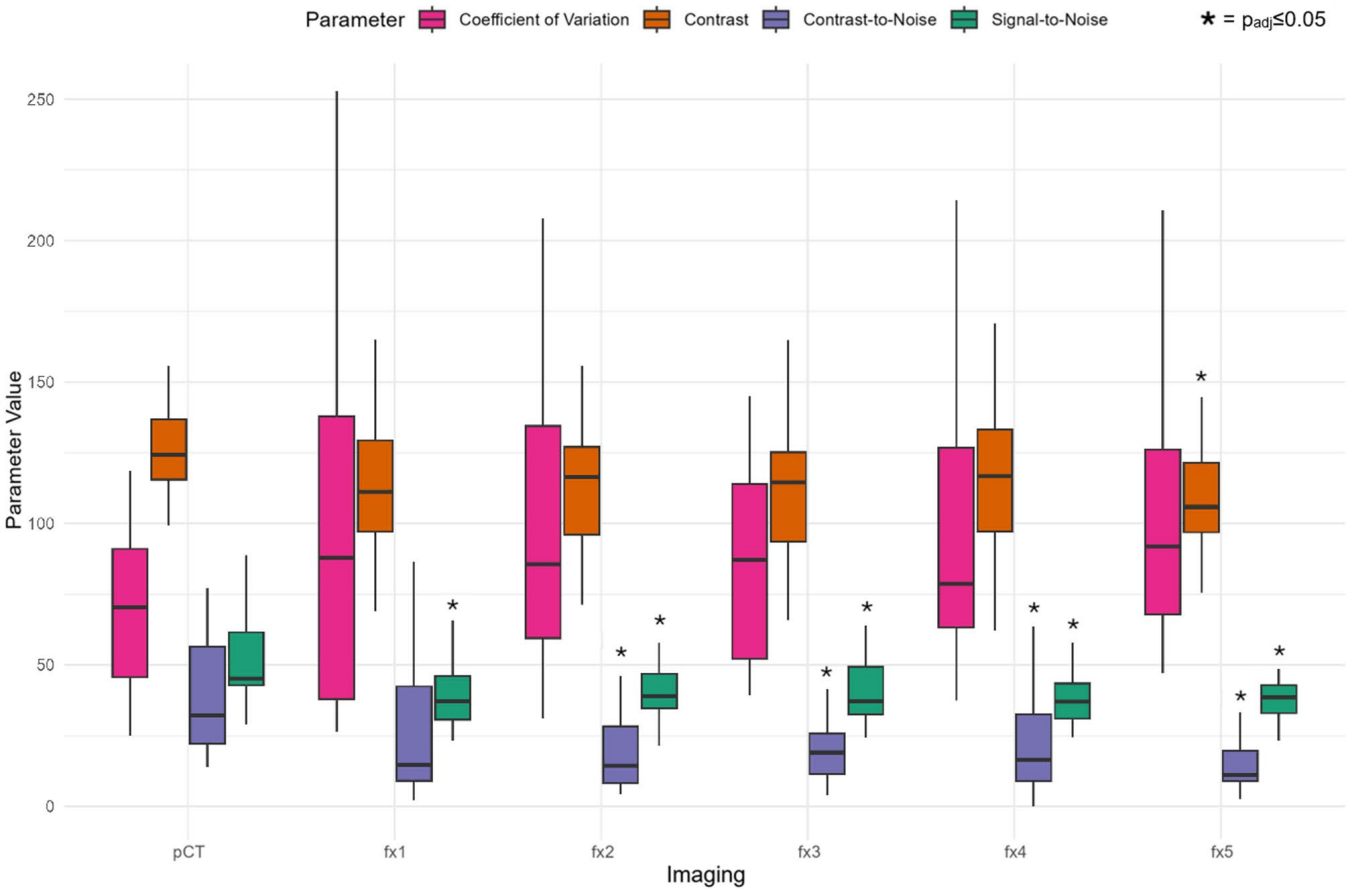
Quantitative Image Quality Parameters in Patient based Analysis of Imaging for adaptive SBRT for prostate cancer. Imaging includes treatment planning computed tomography (pCT) scan (Brilliance BigBore, Philips) with and without metal artefact reduction (MAR) and the five fractional HyperSight^TM^-CBCT scans (fx1-5) with pelvic protocol with and without MAR reconstruction mode on the Ethos^®^ linear accelerator (Varian, Siemens Healthineers). The plots of the quantitative parameters Coefficient of Variation (CV), Signal-to-Noise-Ratio (SNR), contrast (C), contrast-to-noise-ratio (CNR) are given. Asterisks indicate statistical significance in the post-hoc comparison of the quantitative parameters (p_adj_<0.05) between pCT and one of the five fractional HyperSight^TM^-CBCT scans (fx1-5) during adaptive SBRT for prostate cancer. Each dot in the plot represents the mean value of the parameter values for the given imaging stage with the error bars denoting the interquartile range (IQR). The y axis displays the different parameters’ values (unit for C: Hounsfield Unit (HU); for CV: %)

The image quality parameters were significantly different between pCT and fx1-5 (*p* < 0.001) (Fig. 5). For SNR pCT showed higher values compared to fx1-5 (each p_adj_≤0.05), while contrast showed significant differences between pCT and fx5 (p_adj_≤0.05). For CNR pCT showed significant higher values compared to fx2-5 (each p_adj_≤0.05). CV showed no significant differences between pCT and fx1-5.

No significant differences were found between fx1-5 for any parameter.

### Quantitative patient based analysis: longitudinal change of CT attenuation in fractional hCBCT at the target interface

At each fractional hCBCT, the mean CT attenuation (in Hounsfield units) measured in ROI1 was significantly higher than in ROI2 and ROI3 in both regions (each p_adj_≤0.05).

**Fig. 6:**
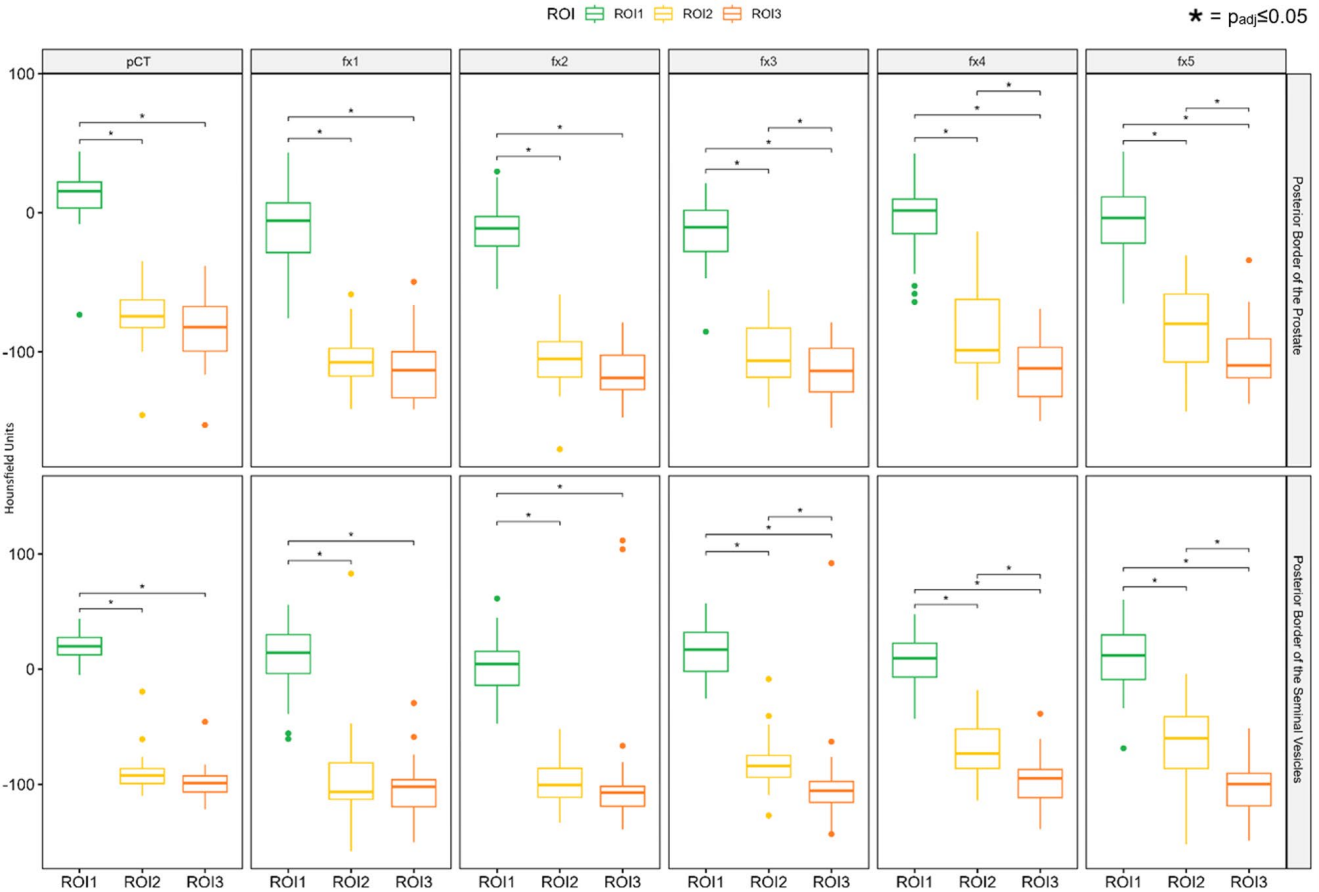
Quantitative parameters in patient based analysis of CT/CBCT-based Imaging for adaptive SBRT for prostate cancer. Imaging includes treatment planning CT (pCT) (Brilliance BigBore, Philips) with and without metal artefact reduction (MAR) and the five fractional HyperSight^TM^-CBCT scans (fx1-5) with pelvis protocol with and without MAR reconstruction mode at the Ethos^®^ linear accelerator (Varian, Siemens Healthineers). Mean Hounsfield Units are being displayed on the y-axis. Boxplots comparing the distribution of measured values across three regions of interest (ROIs) at the target interface (ROI1, ROI2, ROI3, see Fig. 2) for each imaging time point (pCT and fx1–5) in two anatomical regions (posterior border of the prostate, top panels; posterior border of the seminal vesicles, bottom panels). Each box represents the interquartile range (IQR) with the median line, whiskers extend to 1.5×IQR, and outliers are shown as individual points. Pairs of ROIs with statistically significant differences (p_adj_≤0.05) are marked with asterisks

Direct comparisons between ROI2 and ROI3 within the same fraction showed no significant differences at fx1-fx2 but became significantly different at fx3-fx5 with higher CT attenuation values of ROI2 in both regions (each p_adj_≤0.05) (Fig. 6).

When analyzing each ROI longitudinally across fractions, ROI1 and ROI3 showed no significant change over the fractions in either region. In contrast, ROI2 demonstrated significant increases in mean HU across fractions in both regions. (*p* = 0.006 at the prostate, and *p* < 0.001 at the seminal vesicles). Posthoc testing revealed significantly higher CT HU at the prostate in fx5 compared to fx1–2 (each p_adj_≤0.05), and significantly higher values at the seminal vesicles in fx5 compared to fx2, in fx4 compared to fx1-2, and in fx3 compared to fx2 (each p_adj_≤0.05).

Delta(ROI1-ROI2) correlated significantly with the fraction number for both regions (at the prostate CC=-0.2 and at the seminal vesicles CC=-0.4 (each p_adj_≤0.05)). Delta(ROI2-ROI3) correlated also significantly with the fraction number for both regions: at the prostate CC = 0.3 and at the seminal vesicles CC = 0.4 (each p_adj_≤0.05).

## Discussion

This study presents the first longitudinal evaluation of hCBCT for high-precision adaptive SBRT in prostate cancer, combining phantom-based and clinical imaging data. Beyond the technical validation of hCBCT, we demonstrated the clinical robustness of high image quality in both phantom- and patient-based measurements, while evaluating longitudinal soft tissue differentiation.

The hCBCT system itself demonstrated robust technical performance in both phantom‑ and patient‑based analyses, providing stable longitudinal image quality. A repeat phantom assessment performed at a later time point confirmed the stability of the qualitative and quantitative metrics, which corresponded to the stable image quality over time in the patient-based analysis. The consistent image quality across acquisitions supports the feasibility of using hCBCT for ongoing anatomical monitoring in a clinical setting. Over the course of treatment, we were able to demonstrate fraction‑dependent declines in contour sharpness, as well as region-specific changes in CT attenuation. Corresponding decreases in delineation confidence were observed for the ART workflow. These longitudinal changes likely reflect treatment‑related tissue effects and may affect the efficiency of adaptive workflows in daily clinical practice. The demonstrated stable image quality is consistent with the findings of phantom studies by Kim et al. and Lustermanns et al. [11, 17]. Although some variability in the image quality metrics was observed, the system’s performance remains highly reliable for clinical application. These findings further align with those of Haertter et al., who showed that hCBCT achieves image quality comparable to planning CT for treatment planning [18]. hCBCT consistently delivered accurate and reliable imaging, enabling precise, adaptive radiation dose delivery with minimal radiation exposure compared to conventional CT scans [19]. This is a significant advantage especially regarding treatment precision. The slight differences in quantitative and qualitative image quality parameters observed between hCBCT and pCT scans are likely due to the different imaging geometries (fan-beam versus cone-beam-CT) and reconstruction techniques. However, these minor variations did not affect the clinical utility of hCBCT within the cohort presented. This is particularly advantageous, given the typical trade-offs in image quality seen with standard CBCT compared to planning CT scans [20–22]. hCBCT provides significant improvements over standard CBCT, incorporating innovations such as ultra-fast scanning, a larger kilovoltage detector, a thick CsI scintillator, and a reduced grid-to-scintillator distance. These features may position hCBCT, also with iCBCT reconstruction, as a relevant advancement in radiotherapy imaging [23]. However, the technical confirmation of image quality provided by these studies [11, 17–19, 23], and even further confirmed longitudinally by the present study of phantom and patient based data, must be interpreted carefully in the clinical context of patient based imaging, as this does not prevent the radiation oncologist from having to deal with difficult contour delineation, as also shown in the present study.

Prior to ART, the first fractional hCBCT scan showed no significant differences of tissue differentiation and confidence of delineation compared to the planning CT scan, confirming suitability for precise adaptive radiotherapy. However, our study detected a slight longitudinal decrease in organ differentiation and contour sharpness during treatment. This effect was most evident at the organ borders (corresponding to ROI2), where distinction of the fat tissue directly at the interface became less clear. This is likely due to treatment related tissue alterations such as swelling, edema, and mild inflammatory changes at the border of the prostate and seminal vesicles [24, 25]. These therapy related effects may blur the transition between the organ and the surrounding tissue, resulting in a less sharply defined border over time. The fact that ROI1 receives an overall high dose, combined with the fact that ROI2 is localized within a steep gradient of dose exposure, and their different tissue compositions, may lead to distinct imaging responses. Fat tissue may exhibit more pronounced and visible changes in attenuation due to radiation-induced inflammation, increased fluid content, or partial volume effects. In contrast, the signal characteristics of the prostate and seminal vesicles, and the more distant fat tissue ROI3, which is typically outside the high-dose region, remains comparatively stable. Consequently, they appear less affected by at least visible localized treatment-related changes. Taken together, these findings highlight that qualitative and quantitative metrics of tissue visualization may vary based on fraction number, dose exposure, and tissue location. They also underscore the importance of considering these specific anatomical interfaces and local dose exposure when interpreting longitudinal hCBCT data in an adaptive radiotherapy workflow. The results presented could show a prolonged assessment time for delineation confidence, which correlated with the fractional number of the hCBCT. This could indicate that the ultrahypofractionated therapy as it was carried out in this cohort may increasingly lead to more difficult feasibility of adaptive SBRT towards the end of the therapy. Consequently, both different radiotherapy concepts and the robustness of ART need to be re-evaluated in relation to this finding. This finding is also consistent with the results of our previous study on an overlapping cohort regarding the stability of radiomic features during adaptive SBRT [26]. In that study, we demonstrated that robust radiomic features of the entire prostate and their longitudinal stability depend on the number of fractions, which aligns with the findings of this study regarding decreasing contour sharpness and delineation confidence. Interestingly, this contrasts with the quantitative tissue analysis at the prostate’s border, where the mean CT attenuation volumes of specific ROIs in the prostate remain largely stable across treatment fractions. This suggests that radiomic features may be more sensitive to subtle, treatment-induced tissue alterations captured by semi-quantitative reading parameters, but not by mean CT attenuation values of selected ROIs within the prostate. Therefore, radiomic features could serve as a valuable complementary tool for objectively and precisely monitoring tissue changes during high-precision radiotherapy. Future work should explore the correlation between longitudinal radiomic variability and clinically relevant factors such as delineation confidence and adaptation time. Furthermore, the decrease in contour sharpness with stable image quality demonstrated in this study may indicate that clinical margins for target volume definition in high-precision radiation therapy during adaptive SBRT workflows need to be reconsidered. Although we did not directly evaluate the doses absorbed per fraction or target volume boundaries across fractions, the metrics collected on contouring confidence and the time required for evaluation, as well as their correlation with the number of fractions, can serve as indirect indicators of these clinically increasing, relevant effects over the course of the radiation treatment. From a clinical perspective, our study has demonstrated the performance of hCBCT as a possible monitoring tool during high-precision radiotherapy. The potential for even further enhanced image quality using breath-hold techniques - especially for patients with moving targets - underscores its broad applicability [9]. Unlike previous studies, including those by Robar et al., which utilized the iCBCT Acuros mode, our results primarily used the iCBCT reconstruction mode (with partial MAR), which has demonstrated faster reconstruction times while still maintaining robust image quality, being usable for ART as additionally being demonstrated by our study [9]. These findings are relevant, considering that iCBCT has been shown to offer slightly reduced image quality compared to the iCBCT Acuros mode in phantom studies [17, 27]. The combination of high-speed imaging and high-quality images produced by HyperSight™-iCBCT allows for the reduction of treatment adaptation time, thereby demonstrating its advantage in the context of stereotactic ART.

In contrast to an imaging analysis performed by Kunnen et al., who demonstrated the generally increased contour visibility of hCBCT compared to standard CBCT imaging in a cohort of patients undergoing predominantly fiducial based radiotherapy for prostate cancer at an eLinac, this study provides a comprehensive and especially longitudinal analysis from the treatment planning CT, considered the gold standard for target delineation, to each fractional hCBCT-based imaging of the treatment course [28]. Moreover, in contrast to the study by Kunnen et al., the cohort analyzed in the present study was treated with high-precision adaptive radiotherapy rather than standard IGRT and, importantly, without the use of pre-therapeutic fiducial markers, which therefore allowed for a significantly extended and unbiased tissue assessment (without fiducial-related artefacts) of the contours in the prostate/seminal vesicle and rectal border [28]. Based on this combined image quality and anatomical tissue analysis, the presented study was able to further emphasize the findings through the detection of changes in CT attenuation scores at the target interface and also demonstrated a longitudinally decreasing confidence in target delineation with increased time consumption for ART workflow.

This study has several limitations, including the translation of the pre-therapeutic standardized phantom assessments, which have served as an ex vivo baseline for robust image evaluation across observers. However, they showed differences in the SNR between phantom-based and patient-based analyses in pCT and, in comparison, hCBCT examinations. This finding may be due to the different tissues and scattering characteristics that were examined in phantom and patient based scans. The application of MAR and filtered back projection combined with adaptive mAs values in pCT scans, as opposed to CBCT scans, may introduce further relevant bias in quantitative imaging parameters. In addition, the slight, but insignificant variability of the image qualitative parameters in the baseline phantom study must be interpreted carefully, especially with regard to its transferability to patient measurements, since HyperSigh^TM^-CBCT imaging is designed for scanning body constitutions, but quantitative measurements were performed in a flat phantom. Nevertheless, it is relevant here that the stability of image quality in the clinical setting of longitudinal patient measurements could be demonstrated. Further this study used a non-linear CNR metric to enhance low-contrast sensitivity for the specific characteristics of a combined pCT and hCBCT analysis with low-contrast differences in the evaluated tissues. However, this limits direct comparability with studies using linear CNR definitions and may exaggerate the impact of noise in regions with high variance [16, 29]. Regarding the population size of 26 patients, the patient based analysis needs to be validated in a larger cohort. However, adaptive SBRT for prostate cancer represents a highly precise treatment option, and the analysis of all 130 fractional CBCT scans for this study remains the largest homogeneous dataset for hCBCT image quality and anatomical tissue changes to date. As this study was performed at a single institution, a validation study is warranted. hCBCT scans were primarily performed with iCBCT reconstruction; therefore, the Acuros reconstruction mode requires further analysis for image quality and the presented findings of contour sharpness. Scans from three patients were acquired with MAR, which can improve visibility in artefact-affected regions but may alter surrounding tissue appearance, potentially affecting the presented assessments [30]. Further this analysis focused on the pelvis imaging protocol, highlighting the need for translational investigations into different imaging protocols and areas.

Another limitation of this study is that only two observers performed the image evaluations, and one with more experience with adaptive workflows than the other. This approach allowed for the comparison of possible experience‑dependent differences in imaging assessment and usability. Observer experience may have significantly influenced semi-objective image assessments, with hCBCT artefacts and visualization changes appearing more pronounced to less experienced users. However, inter‑ and intra‑observer variability, a potential source of bias, could not be fully captured with such a small number of raters. This is especially true given the limited population size and the systematically higher ratings from the more experienced observer compared to the less experienced observer in our study. Using more raters with comparable expertise may help reduce variability in future studies. It may also allow us to assess the possible dependency of hCBCT assessment on observer experience, as demonstrated by the hypothesis generating study presented here. This further emphasizes the need for more in-depth study of subjective hCBCT interpretation in order to optimize the utility of ART - a topic of particular relevance as departments reevaluate the delineation roles of radiotherapists in ART workflows [10].

Although reading sessions of limited numbers of patients were performed, further observer fatigue must be taken into account as a potential bias. The CBCT datasets were reviewed in chronological order for each patient to correctly attribute changes in image quality and contour sharpness across fractions. Further, the segmentation was carried out in chronological order per patient, which is why external, blinded, and randomized validation should be sought in the future. Another potential bias is tissue changes caused by ADT, which one patient started during ART. ADT may alter prostate volume, impacting daily imaging consistency and CBCT scan assessments [31, 32]. This variability may influence subjective evaluations and complicate analysis of hCBCT image quality. Future studies should stratify patients by ADT status to better understand its impact on prostate size and imaging outcomes. Finally, while hCBCT has shown promising results in prostate cancer SBRT, its efficacy in other tumor sites requiring high soft-tissue contrast remains untested.

As CBCT scans on standard linear accelerators being primarily used for patient positioning, the novel hCBCT imaging represents a significant advancement for clinical practice [10, 33, 34]. hCBCT-equipped hybrid linear accelerators advance precision in radiation oncology by enabling online dose adaptation through superior imaging, improved target delineation (especially critical for re-irradiation to prevent toxicity) [35], and transformation of CBCT from simple guidance to an active adaptive therapy component. With a 100% rate of sufficient and robustly high image quality for ART in this cohort, hCBCT may potentially enable diagnostic monitoring during radiotherapy and even a simulation-free treatment planning approach in radiation oncology becomes feasible. Early reports of simulation-free workflows using hCBCT-based treatment planning highlight its potential to accelerate radiation oncology treatment through CBCT-based adaptive radiotherapy [36]. This integrated imaging and planning approach could streamline workflows, minimize redundant scans, and reduce costs, although additional studies are needed to validate its practicality and safety as a primary planning tool.

## Conclusion

In conclusion, the hCBCT system represents a significant advancement in radiotherapy imaging. While planning CT remains the gold standard for image quality, our data demonstrate the ability of hCBCT to provide equivalent tissue visualization, and detect anatomical changes during adaptive SBRT with a secondary impact on ART workflow. The rating of the observed effects may potentially be influenced by the observer’s experience. Further research is warranted to evaluate the diagnostic potential of hCBCT and its broader application in high-precision radiotherapy with adaptive treatment workflows.

## Data Availability

The data used and generated in this work may be available under ethical and data protection considerations upon request to the leading institution on an individual basis.

## Abbreviations

3D: Three-dimensional
ADT: Androgen deprivation therapy
ART: Adaptive radiotherapy
CBCT: Cone-beam computed tomography
CNR: Contrast-to-Noise-Ratio
C: Contrast
CT: Computed tomography
CV: Coefficient of Variation
eLinac: Ethos^®^ linear accelerator
hCBCT: HyperSight™-CBCT
HU: Hounsfield Unit
iCBCT: Iterative CBCT
ICC: Intraclass Correlation Coefficient
IGRT: Image-guided radiotherapy
IMRT: Intensity-modulated radiotherapy
MAR: Metal artefact reduction
pCT: Planning CT
Pew: Phantom Weighted Error
ROI: Regions of Interest
R1: Observer 1
R2: Observer 2
SBRT: Stereotactic body radiotherapy
SD: Standard deviations
SNR: Signal-to-Noise-Ratio
_C: Central region of the phantom
_F: Pelvic fat
_L: Liver-equivalent inlay
_M: Gluteal muscle
